# Bioelectric Sensors: On the Road for the 4.0 Diagnostics and Biomedtech Revolution

**DOI:** 10.3390/bios10080096

**Published:** 2020-08-11

**Authors:** Spyridon Kintzios

**Affiliations:** Laboratory of Cell Technology, Faculty of Biotechnology, Agricultural University of Athens/EU-CONEXUS European University, 11855 Athens, Greece; skin@aua.gr

Bioelectric sensors lie, by definition, on the interface between biological elements and electronic circuits, irrespective of scale, manufacturing method, and working principle. They distinguish themselves from electrochemical sensors in the sense that they rely exclusively on cells, tissues, and even organs as the biorecognition elements, instead of using only biomolecular moieties, such as antibodies, enzymes, or oligonucleotides.

Bioelectric sensors are quite popular as tools for rapidly accessing the cellular physiologic status: this is a field where both potentiometric and bioimpedance-based biosensors are being increasingly used for toxicity and/or metabolic effects screening [1,2,3,4]. Recent examples in the later application area are represented both by the XF Extracellular Flux Analyzer platform for metabolic assays [5,6] and the Cell Culture Metabolite Biosensor prototype [7] for measuring glycolytic metabolism and inhibitor effects on CD4+ T cells. More advanced systems and approaches are able to provide considerable volumes of experimental information, for example, by means of impedance frequency spectrometry, which can, in turn, be used to train dedicated software for identification and classification of data subgroups. On the biological side, significant progress has been made by immobilizing cells either two-dimensionally onto the surface of conducting electrodes or in a three-dimensional configuration in the appropriate gel: the last option usually contributes to significant simplification and increased efficiency of operation, as well as extended cell viability and storage stability [8].

Among the advantageous traits of bioelectric sensors, speed, non-invasiveness and low cost per assay are the most prominent ones. As a paradigm, bioelectric profiling toxicity assays against pesticide residues can be conducted within a few minutes whereas conventional enzyme-based optical assays may require several hours or even days [9,10]. On the downside, information on the electric properties of living cells and tissues is rarely associated with specific molecular functions, unless the cellular biorecognition element is tailor-made to couple certain biochemical responses to a bioelectric mechanism. Such is the case of membrane-engineered cells and cells with synthetic gene circuits [11,12,13]. Otherwise, the preferable field of application for bioelectric sensors remains that of a more holistic screening of cellular physiology, in particular cell toxicity and membrane channel activity. 

Similar to electrochemical sensors but also distinct from them, bioelectric sensors are able to monitor in real-time, often continuously, physiological patterns and transmit results via Bluetooth/internet to remote data storage, process, and interpretation sites. In several cases, monitoring is conducted non-invasively and, most importantly, not requiring sample extraction. In this way, it is possible to couple biosensors with dedicated, true point-of-care (POC) or point-of-test (POT) platforms (e.g., wearables) that are integrated in various Internet of Things (IoT) networks, including smartphone-based telemetry and e-health applications [14,15,16,17,18,19]. 

In this context, the present Special Issue is not only the first volume exclusively dedicated to bioelectric sensors. In a genuinely emblematic approach, its seven articles, selected through very rigorous peer review and authored by experts of the highest caliber globally, deal with the foremost and advanced technologies and applications in the field of bioelectric sensors. Moreover, they focus on system integration to deliver practical point-of-care/portable and wireless instrumentation and intelligent bioelectric sensing platforms. These will be presented in more detail in following.

Organic biosensors with minimum power consumption represent the next stage of pulse meters, i.e., devices serving as non-invasive rapid medical diagnostic tools by measuring the rate of rhythmic contraction and expansion of an artery, in sync with the heart. They are based on the photoplethysmogram (PPG) principle, according to which, changes in reflected light, detectable as a PPG signal, correspond to changes in the volume of the underlying artery. In their contribution, Elsamnah et al. [20] report the development of a novel organic optoelectronic device purposed as a pulse oximeter and based on two alternative designs using large organic photodiodes (OPDs) and organic light-emitting diodes (OLEDs). These two models were simulated by representing the simplified four-layer structure of a finger model, with red OLED being preferred over green and infrared ones. Both devices were reliable and obtained a clear and stable PPG signal from a healthy individual, with minimum power consumption in wireless monitoring of PPG waveforms. The biosensor pulse meter showed promising results with ultra-low power consumption, 8 µW at 18 dB signal-to-noise ratio (SNR), and demonstrated its ability to measure a clear PPG signal up to 46 dB SNR at a constant current of 93.6 µA. Coupled with a low manufacturing cost, the novel system is very promising for long-term wireless PPG signal monitoring, possibly also as part of a wearable medical device.

Next, Kiani et al. [21] report on the combination of a miniaturized—and therefore fully portable—p-n junction-based Si biochip with impedance spectroscopy, and using the industrial metal-binding, metal-remediating bacteria *Lysinibacillus sphaericus* JG-A12 as the biosensing element. The ohmic or Schottky contacts in the biochip was modelled as the combination of resistors and capacitors, while impedance spectrometry was modelled by using constant phase elements (CPEs). The bulk capacitance of the depletion region of the semiconductor and the capacitance of the Schottky contacts between electrodes and semiconductor contributed to the impedance spectra of the biochips. A linear pattern of response was determined with increasing bacteria concentration measured at test frequencies of 40 Hz, 400 Hz, and 4000 Hz.

Nanotechnology is a major accelerator in the race for continuous device miniaturization and, naturally, bioelectric sensors could not be kept out of this progress. Janssen et al. [22] elaborate on the use of carbon nanotubes (CNTs) for improved sensitivity and response time as potential candidates for PoC protein detection, with the detection of bovine serum albumin (BSA) as a proof-of-concept application. Having a nanometer-scale diameter, CNTs are characterized by large surface and high electrical conductivity, which renders them ideal substrates for manufacturing bioelectric sensing element at the nanoscale. When considering a large, three-dimensional population of such conductive nanoelements interacting with biological moieties—such as antibodies—the systemic conductivity depends on the topological alignment of the nanoelements in this network. In other words, depending on the interaction between nanoelements such as CNTs and their immediate environment, including antibodies and target antigens (analytes in a sample), any disruption of the network continuity will result in a measurable increase in the network’s electrical resistance. This effect is called electrical percolation. The authors applied this working principle to develop a CNT-based, bioelectrical percolation sensor for rapid (10 min) BSA determination with a limit of detection of 2.89 ng/mL and a linear response between 5 and 45 ng/mL. The biosensor was built upon a disposable cellulose paper strip impregnated with CNTs and antibodies for protein detection, the electrical resistance of which was measured with a programmed Arduino Uno.

Application wise, one of the most intriguing and, at the same time, fascinating areas is the intercalation of bioelectric sensors with bioelectromagnetic medical interventions. Wound healing with the aid of external electric fields is such a case. Electrical simulation (ES) is one of the current electromagnetic therapeutic approaches to non-surgical wound healing. Lagoumintzis et al. [23] report on wireless micro current stimulation (WMCS), an alternative non-invasive and non-contact method to electrode-based ES. This approach utilizes the current-carrying capacity of charged air gas, based on the ability of nitrogen (N_2_) and/or oxygen (O_2_) molecules to accept or donate electrons, in order to distribute currents and voltages within the subject tissue. The authors applied an O_2_^−^-induced microcurrent of 1.5–4.0 μA intensity in the patient’s body by using a device capable of producing a specific number of charged particles which covered the wound area from a distance of 12–15 cm. Clinical observations after a three-month treatment period demonstrated the considerable reduction of massive pressure ulcers and the formation of healthy new epithelial tissue. Immunohistochemical analysis revealed both a suppression of inflammation upon WMCS treatment, as well as an increase in myofibroblastic activity, collagen formation, mast cell existence, and a reduced granulocyte aggregation. In essence, the application of tandem WMCS sessions led to reverse the wound-associated electrical leak that short-circuits the skin and to restore the physiological electric fields and ionic currents of the affected tissues. The potential benefits of wide adoption of WMCS in clinical practice as a non-invasive, reagent-free method for wound healing is more than obvious.

Bioelectric profiling is being rapidly established as a superior concept for several applications, including in vitro toxicity, signal transduction, real-time medical diagnostics, environmental risk assessment, and drug development. In the case of cancer, research in the field of hypoxia revealed how critical the pericellular oxygenation in a cell culture is. In this context, a critical marker for the monitoring the differentiation of cancer cells within a cell population is superoxide anion, which is mainly generated as a by-product of the oxidative phosphorylation by the electron transport chain of the mitochondria, is released to the mitochondrial matrix, where it is converted immediately to hydrogen peroxide. Mitochondrial hydrogen peroxide can then diffuse to the cytosol and the nucleus and react with other free radical species, alter signaling pathways or cause cellular damage. Along with other free radical species, superoxide has been found to mediate the development and/or survival of cancer cells and tumors, both in vivo and in vitro [24,25,26]. While hypoxia-regulated processes can result in the bad prognosis of conventional chemotherapy it is essential to monitor and control the cellular microenvironment. Mavrikou et al. [27] demonstrate an innovative and technologically disruptive approach for cell culture monitoring that can be used as an indicator for the response to different chemotherapy options. In particular, they investigated the accumulation of superoxide ions in cultured HeLa cervical cancer cells in response to different 5-fluorouracil (5-FU) concentrations. The anticancer activity of 5-FU emerges from the inhibition of thymidylate synthase (TS) activity during the S phase of the cell cycle and its incorporation into RNA and DNA of tumor cells, as well as from the generation mitochondrial ROS in the p53-dependent pathway [28,29,30,31,32]. Superoxide ion accumulation was monitored with the aid of an advanced bioelectric sensor based on Vero cells which were membrane-engineered with superoxide dismutase. As proven in several reports, the membrane potential of membrane-engineered Vero cell fibroblasts is affected by the interactions of electroinserted SOD molecules and superoxide anions, producing measurable changes in the membrane potential and can be used to determine superoxide extracellular accumulation, e.g., in association with in vitro neuronal differentiation. Therefore, by monitoring superoxide anion concentration in the culture medium after treatment with the chemotherapeutic agent, the authors were able to establish in a high throughput, non-invasive way the in vitro efficacy of 5-FU. This novel cell monitoring tool could be used for the accurate assessment of chemoresistance in cervical and other cancer cells, at least as far as its association with redox balance is concerned [33,34,35].

Within the same field of application and instead of measuring superoxide accumulation in cancer cells, Paivana et al. [36] opted for the direct assessment of the bioelectric properties of four different cancer cell lines (SK-N-SH, HEK293, HeLa and MCF-7) in response, once again, to 5-FU. Cancer cells were immobilized in calcium alginate matrix to mimic the natural tumor environment in vivo and cultured in different cell population densities (50,000 μL, 100,000 μL, and 200,000/100 μL). Bioelectric profiling was conducted by means of bioelectrical impedance-based measurements at three frequencies (1 KHz, 10 KHz, and 100 KHz). For impedance measurements, a voltage of 0.74 Vrms ± 50 mVrms was applied via the two terminals to the gold-coated electrodes. In this way, multi-dimensional mapping (cell line x population density x frequency) was achieved for the response of each cancer cell line against different 5-FU concentrations, in a rapid and entirely non-invasive way. It was demonstrated that bioimpedance measurements were highly correlated with standard cytotoxicity assays. This innovative bioimpedance profiling approach could enable the acquisition of a unique fingerprint for each cancer cell line response to a particular anticancer compound, therefore significantly accelerating the pace of chemotherapy drug screening. 

The final contribution by Ibrahim et al. [37] is the one more closely related to the title of this editorial; namely, the integration of bioelectric sensors in the IoT networks and their role in the ongoing Digital or Industrial Revolution 4.0. In their report, the authors deal with the advanced yet quite an issue of protection against cyberattacks on remote health monitoring systems. In recent years, these systems have experienced almost incredible growth and popularity mainly due to their wide availability as fitness/daily life components of wearables and associated apps. On a more strictly medicinal level, IoT implantable therapeutic equipment and networks (availing over more than one hundred medical tools) are becoming standard issues of modern medical practice. One solution to counter cyberattacks, including tampering, sniffing, and unauthorized access is the construction of attack graphs as a technique to determine risks and vulnerabilities within interoperable systems and to identify possible attack paths. For this purpose, the authors used the pacemaker automatic remote monitoring system (PARMS) as a model for developing tailor-made attack graphs. They illustrate life-threatening risks to patients presented by hacking into the pacemaker’s system and the feasibility of protecting implantable medical devices (IMDs) [38] by carrying out security strategies completely on an external device called a shield. This is definitely a technological field with considerable growth perspectives.

In conclusion, bioelectric sensors are here to stay in spite of their relatively recent emergence in diagnostic technology and related business. Without a doubt, they constitute an internal part of the wearables industry, which will keep on expanding in the next years. Bioelectric profiling is also becoming a valuable tool for rapid toxicity assays and compound x cell type fingerprinting, e.g., in the area of food safety control [10]. Innovative bioelectric sensors are being continuously developed to meet dire and yet unpreceded diagnostic and analytical needs; a vivid, very recent example is the expedient development of a cell-based bioelectric sensor for the ultra-sensitive detection of the SARS-CoV-2 S1 spike protein antigen in just three minutes [39]. As a final comment, bioelectric sensors may evolve as a separate scientific field themselves, opening new perspectives for a deeper understanding of bioelectric phenomena and their exploitation for practical purposes. One of the many possibilities in this direction is demonstrated by the new scientific topic of non-chemical distant cell interaction (NCDCI), where new principles of biology are being currently discovered in parallel with the development of innovative bioelectric sensing tools [40]. 
